# Cytotoxicity of Portuguese Propolis: The Proximity of the *In Vitro* Doses for Tumor and Normal Cell Lines

**DOI:** 10.1155/2014/897361

**Published:** 2014-06-01

**Authors:** Ricardo C. Calhelha, Soraia Falcão, Maria João R. P. Queiroz, Miguel Vilas-Boas, Isabel C. F. R. Ferreira

**Affiliations:** ^1^Centro de Investigação de Montanha (CIMO), ESA, Instituto Politécnico de Bragança, Campus de Santa Apolónia, Apartado 1172, 5301-855 Bragança, Portugal; ^2^Centro de Química, Universidade do Minho, Campus de Gualtar, 4710-057 Braga, Portugal

## Abstract

With a complex chemical composition rich in phenolic compounds, propolis (resinous substance collected by *Apis mellifera* from various tree buds) exhibits a broad spectrum of biological activities. Recently, *in vitro* and *in vivo* data suggest that propolis has anticancer properties, but is the cytoxicity of propolis specific for tumor cells? To answer this question, the cytotoxicity of phenolic extracts from Portuguese propolis of different origins was evaluated using human tumor cell lines (MCF7—breast adenocarcinoma, NCI-H460—non-small cell lung carcinoma, HCT15—colon carcinoma, HeLa—cervical carcinoma, and HepG2—hepatocellular carcinoma), and non-tumor primary cells (PLP2). The studied propolis presented high cytotoxic potential for human tumor cell lines, mostly for HCT15. Nevertheless, excluding HCT15 cell line, the extracts at the GI_50_ obtained for tumor cell lines showed, in general, cytotoxicity for normal cells (PLP2). Propolis phenolic extracts comprise phytochemicals that should be further studied for their bioactive properties against human colon carcinoma. In the other cases, the proximity of the *in vitro* cytotoxic doses for tumor and normal cell lines should be confirmed by *in vivo* tests and may highlight the need for selection of specific compounds within the propolis extract.

## 1. Introduction

Epidemiological data support the concept that natural products, in special, phenols and polyphenols in diet are safe and nontoxic and have long-lasting beneficial effects on human health. Among the many biological properties presented by them, the ones involved with cancer therapy are remarkable [1].

Many plants rich in polyphenols have been shown to display interesting anticancer effects on cell lines and murine models, yielding higher effects when compared to pure natural or synthetic compounds [1]. Products originating in the beehive, such as honey, pollen, royal jelly, and propolis, are attractive both as ingredients for healthy foods and as medicinal products in apitherapy [2, 3].

Propolis is a chemically complex product obtained by bees from the resinous exudates of leaf buds, shoots, and petioles of leaves of different plants present around the hive, which is mixed with wax and salivary secretions. In the hive it has a multifunctional role as a material for construction, maintenance, and defense [4]. Named as bee glue, its chemical composition is highly variable and strongly dependent on the plant sources available around the hive. Furthermore, the propolis composition is dependent on the seasonality, altitude, collector type, food availability, and activity developed during propolis exploitation [5]. More than 300 chemical compounds were identified in propolis from different regions [6, 7]. The main chemical classes and most bioactive compounds found in propolis are the phenolic compounds, including phenolic acids, flavonoids, and their derivatives [6, 8].

Propolis has a wide spectrum of biological and pharmacological properties, which have been intensively researched in cell and animal experiments and thus it can be considered as a drug from the beehive [3]. Recently, studies concerning propolis and cancer have been attracting researchers [9–11]. Propolis and its components, namely, caffeic acid, caffeic acid phenethyl ester (CAPE), 3,5-diprenyl-4-hydroxycinnamic acid (artepillin C), and others, are frequently mentioned in the literature as antitumor and immunomodulatory agents [9, 10, 12]. Through* in vitro* and* in vivo* assays it was possible to define different mechanisms of actions for the bee glue and its components, such as suppressing cancer cells proliferation* via* its anti-inflammatory effects, decreasing the cancer stem cell populations, blocking specific oncogene signaling pathways, exerting antiangiogenic effects, and modulating the tumor microenvironment [9–11].

Propolis phenolic profile from different Portuguese regions was recently characterized and quantified through high-pressure liquid chromatography (HPLC) and liquid chromatography with diode-array detection coupled to electrospray ionization tandem mass spectrometry (LC/DAD/ESI − MS^ n ^), providing identification of seventy-six phenolic compounds [13, 14]. Within the biological properties studied in Portuguese propolis, few studies deal with anticancer effects. Two samples from the northeast and center of Portugal strongly suppress the proliferation of primary renal cancer cells in a concentration-dependent manner [15]. Also, the* in vitro* antitumor activity of a propolis sample from Azores archipelago was evaluated on colon carcinoma cell line HCT15 presenting cytotoxicity in a dose- and time-dependent way [16].

Due to the complex nature of propolis which strongly depends on the local flora at the site of collection, for an effective evaluation of the anticancer properties of Portuguese propolis more regions have to be evaluated and the antitumor assays should be extended for other types of cell lines. Moreover, it is important to compare its cytotoxicity against tumor and normal cells. In this context, this work aims to study the growth inhibitory activity of phenolic extracts from Portuguese propolis of different regions on human tumor cell lines (MCF-7—breast adenocarcinoma, NCI-H460—non-small cell lung carcinoma, HCT15—colon carcinoma, HeLa—cervical carcinoma, and HepG2—hepatocellular carcinoma) and nontumor primary cells (PLP2).

## 2. Material and Methods

### 2.1. Samples

#### 2.1.1. Samples Origin

The study was performed on propolis samples collected from five different geographical regions in Portugal: north (N1, Bragança; N8, Mirandela), central coast (CC3, Coruche), south (S2, Aljezur), Azores Archipelago (A2, S. Miguel Island), and Madeira Island (M1, Funchal). All the samples were obtained between 2007 and 2009 after the honey harvesting season (July/September), by conventional scraping or through plastic screens. These propolis samples were then stored at −20°C until analysis.

#### 2.1.2. Phenolic Extracts Preparation

The extraction was made according to the work previously described [8]. Prior to the extraction, 1 g of powdered propolis sample was homogenized and mixed with 10 mL of 80% of ethanol/water and kept at 70°C for 1 h. The resulting mixtures were filtered and the residues were reextracted in the same conditions. After the second extraction, the filtrates were combined, concentrated, and freeze-dried. Complete chemical characterization of the phenolic extracts is available in Falcão et al. [13, 14] For cytotoxicity evaluation, the extracts were dissolved in DMSO at a final concentration of 8 mg/mL. The final solutions were further diluted to different concentrations. The results were expressed in GI_50_ (extract concentration that inhibited 50% of the net cell growth) values. Ellipticine was used as positive control.

### 2.2. Standards and Reagents

Fetal bovine serum (FBS), L-glutamine, Hank's balanced salt solution (HBSS), trypsin-EDTA (ethylenediaminetetraacetic acid), penicillin/streptomycin solution (100 U/mL and 100 mg/mL, resp.), RPMI-1640, and DMEM media were from Hyclone (Logan, USA). Acetic acid, ellipticine, sulforhodamine B (SRB), trypan blue, trichloroacetic acid (TCA), and Tris were from Sigma Chemical Co. (Saint Louis, USA). Water was treated in a Milli-Q water purification system (TGI Pure Water Systems, USA).

### 2.3. Evaluation of Cytotoxicity in Human Tumour Cell Lines 

Five human tumor cell lines were used: MCF-7 (breast adenocarcinoma) from DSMZ (Leibniz-Institut DSMZ—Deutsche Sammlung von Mikroorganismen und Zellkulturen GmbH), NCI-H460 (non-small cell lung carcinoma), HCT15 (colon carcinoma), HeLa (cervical carcinoma), and HepG2 (hepatocellular carcinoma) from European collection of cell cultures (ECACC). Cells were routinely maintained as adherent cell cultures in RPMI-1640 medium containing 10% heat-inactivated FBS (MCF-7, NCI-H460, and HCT15) and 2 mM glutamine or in DMEM supplemented with 10% FBS, 2 mM glutamine, 100 U/mL penicillin, and 100 mg/mL streptomycin (HeLa and HepG2 cells), at 37°C, in a humidified air incubator containing 5% CO_2_. Each cell line was plated at an appropriate density (7.5 × 10^3^ cells/well for MCF-7, NCI-H460 and HCT15 or 1.0 × 10^4^ cells/well for HeLa and HepG2) in 96-well plates for 24 h. Cells were then treated for 48 h with various extract concentrations. Following this incubation period, the adherent cells were fixed by adding cold 10% trichloroacetic acid (TCA, 100 *μ*L) and incubated for 60 min at 4°C. Plates were then washed with deionized water and dried; sulforhodamine B solution (0.1% in 1% acetic acid, 100 *μ*L) was then added to each plate well and incubated for 30 min at room temperature. Unbound SRB was removed by washing with 1% acetic acid. Plates were air-dried, the bound SRB was solubilised with 10 mM Tris (200 *μ*L) and the absorbance was measured at 540 nm in ELX800 Microplate Reader (Bio-Tek Instruments, Inc; Winooski, USA) [17].

### 2.4. Evaluation of Cytotoxicity in a Porcine Liver Primary Cell Culture

A cell culture was prepared from a freshly harvested porcine liver obtained from a local slaughter house, and it was designed as PLP2. Briefly, the liver tissues were rinsed in Hank's balanced salt solution containing 100 U/mL penicillin and 100 *μ*g/mL streptomycin and divided into 1 × 1 mm^3^ explants. Some of these explants were placed in 25 cm^2^ tissue flasks in DMEM medium supplemented with 10% fetal bovine serum, 2 mM nonessential amino acids, 100 U/mL penicillin, and 100 mg/mL streptomycin and incubated at 37°C with a humidified atmosphere containing 5% CO_2_. The medium was changed every two days. Cultivation of the cells was continued with direct monitoring every two to three days using a phase contrast microscope. Before confluence was reached, cells were subcultured and plated in 96-well plates at a density of 1.0 × 10^4^ cells/well and cultivated in DMEM medium with 10% FBS, 100 U/mL penicillin, and 100 *μ*g/mL streptomycin [17].

### 2.5. Statistical Analysis

For all the experiments three samples (*n* = 3) were analysed and all the assays were carried out in triplicate. The results are expressed as mean values and standard deviation (SD). The differences between the different samples were analysed using one-way analysis of variance (ANOVA) followed by Tukey's honestly significant difference post hoc test with *α* = 0.05, coupled with Welch's statistic. This treatment was carried out using SPSS v. 18.0 program. The regression analysis between phenolic acids, phenolic esters, total phenolics, flavonols, flavones, dihydroflavonols, flavanones, flavonoid esters, total flavonoids (mg/g of extract), and cytotoxicity GI_50_ values (*μ*g/mL) was performed using the same statistical package.

## 3. Results

**Figure 1:**
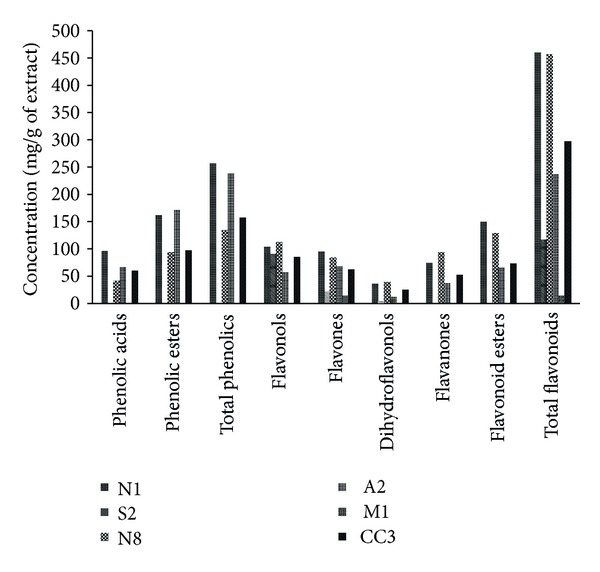
Phenolic classes presented in propolis of different Portuguese origins. [14].

The cytotoxicity of the propolis phenolic extracts was evaluated in five human tumor cell lines (breast- MCF7, lung- NCI-H460, colon- HCT15, cervical- HeLa and hepatocellular- HepG2, carcinomas) and in a porcine liver primary cell culture (PLP2), established by us. All the studied extracts inhibited the growth of the mentioned tumor cell lines. N1 gave the highest cytotoxicity, followed by A2 (Table 1), presenting the lowest GI_50_ values against the tested tumor cell lines. The HCT15 cell line was the most sensible to the studied extracts, except to S2 extract, as the A2 extract was the most active (GI_50_ = 9 *μ*g/mL; an excellent GI_50_ value in comparison with several natural extracts, for example, Guimarães et al. [17] This activity could be related to the chemical composition of this sample (Figure 1). From the analysis of Figure 1, it can be observed that samples N1 and A2 have major concentrations of total phenolics and phenolic acids, especially the sample N1.

Despite the lower concentration in total phenolics presented in the sample N8, the GI_50_ values for MCF7 cell line were not very different from the extracts N1 or A2, which can be due to the similar high content in total flavonoids and the specificity of this cell line to flavonoids. This sample presented the major concentration of flavones and flavanones, two important groups of compounds regarding biological activity.

The M1 sample from Madeira island showed the highest GI_50_ values for all the tested tumor cell lines. This fact could be explained by its poor chemical composition (Figure 1) that only includes flavones and in low concentration.

The correlations established between the chemical composition and the cytotoxicity results are presented in Table 2. The cytotoxicity observed for all the tested tumor cell lines, except MCF7, was positively correlated with phenolic acids (*R* ^2^ values higher than 0.5) and, consequently, with total phenolic (*R* ^2^ higher than 0.5) contents but not with phenolic esters (*R* ^2^ lower than 0.5). NCI-H460 cell line presented the highest correlation for this class of compounds (*R* ^2^ = 0.9454).

Total flavonoids were positively correlated (*R* ^2^ values higher than 0.5) with the cytotoxicity observed against MCF7, NCI-H460, HCT15, and HeLa cell lines. However, the cytotoxicity was not correlated (*R* ^2^ values lower than 0.5) with flavonols, dihydroflavonols, and flavonoid esters. The cytotoxicity observed in HCT15 cell line was the one that correlates better with the composition in phenolic compounds.

The evaluation of the cytotoxicity using PLP2 is very important since mammalian hepatocytes still represent an obligatory step in the evaluation of toxic compounds that lead to the production of various metabolites, which are the ultimate cause of toxicity. We used porcine liver as an* in vitro* cytotoxicity model because it is known, in terms of cellular and physiological functioning, to be very similar to human. Despite the high cytotoxicity displayed by most of the propolis samples against tumor cell lines, the samples also showed toxicity for nontumour (normal) liver primary culture (PLP2), with GI_50_ values close to those obtained for tumor cell lines. The cytotoxicity for PLP2 primary culture was only positively correlated with phenolic acids (*R* ^2^ = 0.7442) and consequently with total phenolics (*R* ^2^ = 0.6207).

The colon carcinoma cells, HCT15, seem to be the most promising to be controlled by the studied propolis extracts since, with exception of sample S2, all the cytotoxicity levels for the tumor cells are significantly above the ones found for nontumor PLP2 cells. The same behaviour was observed for the NCI-H460 cell line; however, in this case the cytotoxicity levels for tumor and nontumor cells are close which reduces the selectivity. The sample from Madeira showed the lowest cytotoxicity for all the tested tumor cell lines, which indicates that the chemical composition of the samples has a significant influence on their bioactivity, since this sample presented a quite specific phenolic profile, only with flavones.

## 4. Discussion 

The highest cytotoxicity revealed by samples N1 and A2 against all the tested tumor cell lines is certainly related to their higher concentrations in total phenolics and phenolic acids. Phenolic acids and analogues are, in fact, known to display a wide variety of biological functions, in addition to their primary antioxidant activity, which are mainly related to modulation of carcinogenesis [18, 19]. Indeed, many phenolic compounds have been investigated for their potential use as cancer chemopreventive agents [1, 20]. There are many reports on the bioactive constituents of propolis like cinnamic acid esters, such as caffeic acid phenethyl (CAPE), which is a component of propolis from honeybee hives, and benzyl esters that display selective antiproliferative, anticarcinogenic, and immunomodulatory properties [21, 22]. The molecular basis of this action may be connected to the inhibition of the nuclear transcription factor NK-kappa B [23]. Other authors refer that drupanin, baccharin, cinnamic acid derivatives inhibit the growth of HeLa-60 cell lines by inducing apoptosis [24].

The similar toxicity for MCF7 cell line observed between N8, N1, and A2 can be explained by the similar high content in total flavonoids, presenting N8 as the highest concentration of flavones and flavanones. The most common compounds within the flavones were apigenin, chrysin, and luteolin. They are responsible for main benefits to human health including anticarcinogenic effects [25]. In the flavanones group, hesperidin and naringin, the most important compounds, have also been reported as possessing anticancer properties [25].

The present data highlights the high cytotoxicity of Portuguese propolis against tumor cell lines, being in agreement with Búfalo et al. [26], who reported a marked activity of Brazilian green propolis against other tumor cells (human laryngeal epidermoid carcinoma (HEp-2)); the main mechanism seems to involve apoptosis [27]. Ethanol extract of Brazilian red propolis was also reported as promoting a significant reduction in MCF7 cell viability thought the induction of mitochondrial dysfunction, caspase-3 activity, and DNA fragmentation [28]. Moroccan propolis extracts (ethanolic and ethyl acetate) were described as exerting antiproliferative effect against BSR (hamster renal adenocarcinoma), Hep-2 (Human laryngeal carcinoma), and P815 (murine mastocytoma) in dose-dependent manner [29]. Similar results were obtained by Sulaiman et al. [30] with Iraqi propolis, who described the inhibitory effects against the proliferation of HL-60 and colony potential of HCT116 cells.

Despite the high cytotoxicity displayed by most of the propolis samples against tumor cell lines studied herein, the samples also showed toxicity for nontumour (normal) liver primary culture (PLP2). da Silva Frozza et al. [31] have also investigated the cytotoxicity of extracts of red Brazilian propolis for two tumor cell lines (Hep-2 and HeLa) and human normal epithelial embryonic kidney (Hek-293), reporting also higher IC_50_ value for Hek-293 when compared to tumor cell lines.

## 5. Conclusions

Propolis phenolic extracts comprise phytochemicals that should be further studied for their bioactive properties against human colon carcinoma. In the other cases, the proximity of the* in vitro* cytotoxic doses for tumor and normal cell lines should be taken into consideration and confirmed by* in vivo* tests. Additional research is needed to determine which compounds are involved in cytotoxicity against normal cells in order to develop new propolis formulations without those compounds to be selectively used against tumor cells.

## Figures and Tables

**Table 1:** Cytotoxicity of phenolic extracts from Portuguese propolis of different origins (mean ± SD).

	MCF-7 (breast carcinoma)	NCI-H460 (non-small lung cancer)	HCT15 (colon carcinoma)	HeLa (cervical carcinoma)	HepG2 (hepatocellular carcinoma)	PLP2 (non-tumour liver primary culture)
A2	39 ± 0	29 ± 2^c^	9 ± 1^d^	38 ± 4^d^	34 ± 4^bc^	35 ± 3^d^
CC3	37 ± 2^d^	30 ± 0^c^	12 ± 1^d^	40 ± 3^d^	34 ± 2^bc^	36 ± 1^d^

M1	182 ± 2^a^	166 ± 6^a^	120 ± 3^a^	182 ± 11^a^	153 ± 6^a^	180 ± 3^a^
N1	36 ± 1^d^	23 ± 1^d^	10 ± 1^d^	38 ± 3^d^	30 ± 3^c^	29 ± 3^d^
N8	41 ± 1^c^	34 ± 2^bc^	38 ± 5^c^	54 ± 4^c^	40 ± 3^b^	54 ± 7^b^
S2	47 ± 2^b^	37 ± 1^b^	50 ± 11^b^	84 ± 6^b^	36 ± 2^bc^	45 ± 1^c^

Ellipticine	0.91 ± 0.04	1.42 ± 0.00	1.91 ± 0.06	1.1 ± 0.2	3.2 ± 0.7	2.06 ± 0.03

GI_50_ values (*μ*g/mL) corresponding to the sample concentration achieving 50% of growth inhibition in human tumor cell lines or in liver primary cultured PLP2.

In each column different letters mean significant differences (*P* < 0.05).

**Table 2:** Correlations established between content in phenolics and flavonoids (mg/g of extract) and cytotoxicity GI_50_ values (*μ*g/mL) of phenolic extracts from Portuguese propolis of different origins.

	MCF7	NCI-H460	HCT15	HeLa	HepG2	PLP2
Phenolic acids	*y* = −6.9987*x* + 333.66 *R*² = 0.4883	*y* = −4.7627*x* + 204.52 *R*² = 0.9454	*y* = −1.1696*x* + 86.11 *R*² = 0.5367	*y* = −2.1202*x* + 156.37 *R*² = 0.5928	*y* = −4.1673*x* + 209.11 *R*² = 0.7655	*y* = −1.7531*x* + 133.46 *R*² = 0.7442
Phenolic esters	*y* = −3.3612*x* + 259.49 *R*² = 0.034	*y* = −6.0297*x* + 306.39 *R*² = 0.4579	*y* = −1.882*x* + 163.43 *R*² = 0.4201	*y* = −3.4135*x* + 276.56 *R*² = 0.4643	*y* = −4.9099*x* + 299.63 *R*² = 0.3211	*y* = −2.4935*x* + 226.99 *R*² = 0.4549
Total phenolics	*y* = −10.36*x* + 593.15 *R*² = 0.1521	*y* = −10.792*x* + 510.91 *R*² = 0.6900	*y* = −3.052*x* + 249.54 *R*² = 0.5195	*y* = −5.5337*x* + 432.93 *R*² = 0.5740	*y* = −9.0772*x* + 508.74 *R*² = 0.5163	*y* = −4.2466*x* + 360.45 *R*² = 0.6207

Flavonols	*y* = 0.0481*x* + 87.45 *R*² = 0.0001	*y* = 0.3793*x* + 77.724 *R*² = 0.0092	*y* = 0.4697*x* + 78.163 *R*² = 0.1837	*y* = 0.2617*x* + 76.058 *R*² = 0.0587	*y* = 1.1011*x* + 51.202 *R*² = 0.0471	*y* = 0.9117*x* + 53.017 *R*² = 0.1874
Flavones	*y* = −0.3895*x* + 82.467 *R*² = 0.4736	*y* = −0.4097*x* + 79.497 *R*² = 0.4786	*y* = −0.591*x* + 81.208 *R*² = 0.5913	*y* = −0.4634*x* + 91.432 *R*² = 0.6394	*y* = −0.4459*x* + 81.911 *R*² = 0.4314	*y* = −0.3797*x* + 81.606 *R*² = 0.4507
Dihydroflavonols	*y* = −2.1673*x* + 109.73 *R*² = 0.4027	*y* = −1.2445*x* + 61.239 *R*² = 0.1981	*y* = −0.196*x* + 27.711 *R*² = 0.0645	*y* = −0.3877*x* + 42.748 *R*² = 0.2585	*y* = 0.0286*x* + 22.028 *R*² = 0.0006	*y* = 0.458*x* + 10.091 *R*² = 0.1726
Flavanones	*y* = 2.4455*x* − 29.308 *R*² = 0.0469	*y* = 1.0683*x* + 33.159 *R*² = 0.0374	*y* = 1.3164*x* + 41.508 *R*² = 0.5347	*y* = 2.0971*x* − 25.231 *R*² = 0.4561	*y* = 1.719*x* + 5.1786 *R*² = 0.1024	*y* = 1.4251*x* + 9.3367 *R*² = 0.3868
Flavonoid esters	*y* = −1.6488*x* + 167.17 *R*² = 0.0079	*y* = −2.4757*x* + 176.14 *R*² = 0.0745	*y* = 1.1061*x* + 84.981 *R*² = 0.1401	*y* = 1.6454*x* + 33.882 *R*² = 0.1042	*y* = −0.4261*x* + 118.74 *R*² = 0.0023	*y* = 0.5517*x* + 82.834 *R*² = 0.0215
Total flavonoids	*y* = −2.2*x* + 403.65 *R*² = 0.5071	*y* = −2.3009*x* + 386.17 *R*² = 0.5066	*y* = −3.05*x* + 383.97 *R*² = 0.5191	*y* = −2.4582*x* + 442.68 *R*² = 0.6038	*y* = −2.5236*x* + 400.76 *R*² = 0.4636	*y* = −2.0844*x* + 395.37 *R*² = 0.4529
